# Comparative Analgesic Efficacy of Diclofenac and Ketoprofen Transdermal Patches Versus Ibuprofen Tablets for Pain Control After Initial Archwire Placement in Orthodontic Patients: A Randomized Clinical Trial

**DOI:** 10.1155/prm/9922008

**Published:** 2026-05-22

**Authors:** Yasamin Babaee Hemmati, Amirhossein Toghrolian

**Affiliations:** ^1^ Department of Orthodontics, Dental Sciences Research Center, School of Dentistry, Guilan University of Medical Sciences, Rasht, Iran, gums.ac.ir; ^2^ Department of Orthodontics, School of Dentistry, Isfahan University of Medical Sciences, Isfahan, Iran, mui.ac.ir

**Keywords:** diclofenac, fixed, ketoprofen, orthodontic appliances, transdermal patch

## Abstract

**Objectives:**

This study compared the analgesic efficacy of diclofenac and ketoprofen transdermal patches versus ibuprofen tablets for pain control following initial archwire placement in orthodontic patients.

**Materials and Methods:**

In this randomized clinical trial, 60 patients (aged 15–30 years) undergoing fixed orthodontic treatment (0.022‐inch MBT brackets and 0.014‐inch nickel–titanium archwires on both arches) were assigned via stratified permuted block randomization (1:1:1 ratio) to three groups (*n* = 20 each): 30‐mg ketoprofen transdermal patches every 12 h, 15‐mg diclofenac transdermal patches every 12 h, or 400‐mg ibuprofen tablets every 8 h for 1 day. Pain severity was assessed using the numeric rating scale (NRS; 0–10) at baseline (immediately postplacement), 2 and 6 h, bedtime, 24 and 48 h, and 3 and 7 days. Data were analyzed by the generalized estimating equation (GEE) and the Kruskal–Wallis, Bonferroni, chi‐square, and Fisher’s exact tests (*α* = 0.05).

**Results:**

The pain score was the lowest in the ketoprofen group, followed by the ibuprofen group, and then the diclofenac group, but the difference was not significant (*p* > 0.05). The pain score initially increased after archwire placement to 6 h (from 0 pain in all groups to 2.50 ± 1.64, 3.90 ± 1.25, and 3.45 ± 1.82, respectively, in ketoprofen, diclofenac, and ibuprofen groups). It subsequently decreased until bedtime, increased again at 24 h, and then followed a descending trend from 48 h to 7 days. The trend of change in the pain score over time was not significantly different among the three groups (*p* = 0.657). Age (*p* = 0.757) and gender (*p* = 0.153) of patients did not affect their perceived pain.

**Conclusion:**

Considering the comparable analgesic efficacy of ibuprofen and the tested transdermal patches, diclofenac and ketoprofen patches may be used as an alternative to ibuprofen tablets for efficient pain control after initial archwire placement.

**Trial Registration:** Iranian Registry of Clinical Trials: IRCT20190915044771N2

## 1. Introduction

Orthodontic treatment is performed to correct the position of misaligned teeth and create a beautiful smile using archwires, bonded brackets, aligners, or other orthodontic appliances for orthodontic tooth movement. However, this process can be associated with pain and discomfort for the patients. Understanding and management of this pain can facilitate the course of treatment for patients [1].

Pain is an unpleasant sensory experience associated with actual or possible tissue injury [2]. Pain control is highly important in orthodontic treatment since pain is the most common patient complaint and a major factor adversely affecting patient compliance [3]. The prevalence of orthodontic pain is relatively high, ranging from 77% to 95% [3, 4]. Orthodontic pain is caused by the ischemia, inflammation, and edema of the compressed periodontal ligament. Inflammatory mediators such as histamine, bradykinin, prostaglandins, serotonin, and substance P are released in an inflamed periodontal ligament. These mediators stimulate the pain receptors and generate pain as such [5]. Orthodontic pain often initiates within 2 h following load application, reaches its peak during sleep or within 24 h after appliance placement, and lasts for 5–7 days [6]. Orthodontic pain depends on factors such as age, gender, personal pain threshold, magnitude of the applied force, emotional state and stress level of patient, cultural issues, and previous pain experiences [7].

Different methods have been proposed for orthodontic pain control such as pharmacotherapy with nonsteroidal anti‐inflammatory drugs (NSAIDs), low‐level laser therapy (LLLT) [5], transcutaneous electrical nerve stimulation [8], vibratory stimulation of the periodontal ligament, and bite wafers [6]. To date, using NSAIDs has been the most successful method of orthodontic pain control and is considered as the gold standard for this purpose [6, 9]. In brief, NSAIDs inhibit the cyclooxygenase (COX) enzymes and prevent the synthesis of arachidonic acid and subsequently the prostaglandins, which are important pain mediators [10]. Ibuprofen and diclofenac sodium are the most popular NSAIDs used for orthodontic pain control due to their optimal analgesic and anti‐inflammatory properties [11].

Ketoprofen is another NSAID that not only inhibits the COX and lipoxygenase pathways but also causes central and peripheral desensitization. Unlike other NSAIDs, ketoprofen inhibits central prostaglandin synthesis at both peripheral and central levels (brain COX and nitric oxide synthase) and is therefore expected to show high analgesic efficacy [12]. The half‐life of ibuprofen is 1.8–2 h, while the half‐life of diclofenac is 2 h [13] and that of ketoprofen is 2–2.5 h [14]. Close half‐life of these medications enables a more accurate comparison of their analgesic efficacy.

Analgesics can be used through oral, injection, inhalation, and transdermal routes. Oral administration of a drug carries the potential for its first‐pass metabolism and loss of a significant amount of the drug before it is absorbed systemically [15]. Moreover, oral intake of medications can be associated with a risk of gastrointestinal problems, renal insufficiency, hepatotoxicity, aggravation of asthma, sodium retention, hypertension, and resistance to antihypertensive medications due to the high plasma concentration of the drug [16]. The injection route is also painful and can cause a sudden increase in the plasma level of the drug and the associated side effects or complications [15].

Transdermal patches are medicated adhesive patches placed on the skin to deliver a certain dose of medication through the skin portals directly to the bloodstream [17]. Not undergoing the first‐pass metabolism in the liver, reduced plasma concentration, and subsequently lower systemic toxicity and side effects are the main advantages of transdermal patches. In addition, they are better accepted by patients [18] and can be particularly beneficial for patients with trypanophobia [19].

Several studies have evaluated the analgesic efficacy of NSAID transdermal patches for pain control following tooth extraction [18–21], periodontal surgery [22–24], maxillofacial surgical procedures [25], and endodontic treatment [26–29]. However, to the best of the authors’ knowledge, the efficacy of transdermal patches for orthodontic pain control has not been previously investigated. In addition, controversy exists regarding the efficacy of transdermal patches. Three studies in the field of periodontology and pain control after periodontal flap surgery compared the analgesic efficacy of diclofenac sodium tablets and transdermal patches and showed that the patches had higher, equal, or lower analgesic efficacy than tablets [22–24]. Studies on pain control after tooth extraction also showed that the diclofenac transdermal patches had equal [19, 30] or slightly lower efficacy than tablets in the first 24 h after the procedure [15, 22]. Additionally, diclofenac transdermal patches can have equal or lower analgesic efficacy compared with ketoprofen patches [18, 21]. Studies on the efficacy of transdermal patches for endodontic pain control also showed almost equal analgesic efficacy of diclofenac tablets and patches, as well as ketoprofen patches and ibuprofen tablets [26, 31].

Considering the significance of orthodontic pain control with minimal complications and side effects, this study aimed to compare the analgesic efficacy of diclofenac and ketoprofen transdermal patches versus ibuprofen tablets for pain control following initial archwire placement in orthodontic patients. The null hypothesis of the study was that no significant difference would be found in the analgesic efficacy of diclofenac and ketoprofen transdermal patches versus ibuprofen tablets for pain control following initial archwire placement in orthodontic patients.

## 2. Materials and Methods

This study was conducted at the Orthodontics Department of School of Dentistry, Guilan University of Medical Sciences from April 2024 to November 2024. The study protocol was approved by the ethics committee of the university (IR.GUMS.REC.1402.577). The registration is available at

 IRCT: https://irct.behdasht.gov.ir/trial/75633 (note: access may be restricted from some locations)
 WHO ICTRP: https://trialsearch.who.int/Trial2.aspx?TrialID=IRCT20190915044771N2.

### 2.1. Trial Design

This was a randomized, parallel‐group clinical trial designed to detect differences between the treatments. Patients received either diclofenac transdermal patches, ketoprofen transdermal patches, or ibuprofen tablets for pain control following initial archwire placement. The trial was analyzed on an intention‐to‐treat basis. The results are reported in accordance with the Consolidated Standards of Reporting Trials (CONSORT).

### 2.2. Participants, Eligibility Criteria, and Settings

The inclusion criteria were patients requiring fixed orthodontic treatment of both the maxillary and mandibular arches; age range of 15–30 years; physical health with no systemic disease (ASA I); the presence of 3–6 mm of crowding in both the maxillary and mandibular arches with no spacing and no need for tooth extraction according to Little’s Irregularity Index [32]; no medication intake; no history of allergy, gastrointestinal bleeding, or discomfort following the intake of aspirin‐like medications such as ibuprofen (NSAIDs in general); and having no problem taking ketoprofen, diclofenac, or ibuprofen.

The exclusion criteria were patients who had taken analgesics during 24 h prior to the treatment onset; illiterate patients (inability to read and write); those requiring restorative, endodontic, or extraction treatments; patients with spontaneous toothache or periodontal problems contraindicating orthodontic treatment; patients not willing to participate in the study; pregnancy or nursing; and those with a history of renal or hepatic diseases, or taking medications affecting the kidneys or the liver. In addition, patients who did not take/use the assigned medication at the required time points according to the provided timetable or did not correctly report their pain score at the designated time points, patients with one or more brackets debonded in the first week after the treatment onset, and patients who required drug or analgesic intake for nonorthodontic reasons during the first week after the treatment onset were all excluded.

The sample consisted of 60 patients between 15 and 30 years requiring fixed orthodontic treatment who were selected among those presenting to the Orthodontics Department of School of Dentistry, Guilan University of Medical Sciences, during the aforementioned time period.

### 2.3. Interventions

Written informed consent was obtained from all eligible participants prior to their enrollment. The consent form included important information about the study objectives, methodology, and advantages and possible side effects of medications. The patients were scheduled to undergo fixed orthodontic treatment at the Orthodontics Clinic of School of Dentistry of Guilan University of Medical Sciences by postgraduate students of orthodontics or orthodontists.

Demographic information of patients was initially extracted from their records. All patients received orthodontic appliances including a buccal tube on first molars, brackets (0.022‐inch MBT system), and 0.014‐inch nickel–titanium archwires on both dental arches during a morning session. The patients were then randomly assigned to three parallel groups (*n* = 20) with 1:1:1 allocation ratio using permuted blocks of size 3 (one allocation each for ketoprofen, diclofenac, and ibuprofen):

 A: Patients received 30 mg ketoprofen transdermal patches (Kefentech Plaster 30 mg, Jeil Health Science, Yongin‐Si, South Korea) immediately after archwire placement and also after 12 and 24 h.
 B: Patients received 15 mg diclofenac transdermal patches (Defen Plaster 15 mg, Sinil Pharmaceutical Co, Seoul, Republic of Korea) immediately after completion of archwire placement and also after 12 and 24 h.
 C: Patients received 400 mg ibuprofen tablets (RAHA Pharmaceutical Co., Tehran, Iran) immediately after archwire placement and 8, 16, and 24 h later.

The abovementioned transdermal patches contain the lowest available dose internationally and also in Iran. Ibuprofen was used as the gold standard for the reduction of orthodontic pain [33, 34].

To promote adherence, each patient received a printed timetable outlining the exact dosing schedule along with contact instructions. The researcher provided verbal reinforcement of the schedule during the initial dispensing and confirmed the first dose was administered on‐set after completion of bracket bonding and activation of #14 nickel–titanium archwire. Compliance was monitored through scheduled follow‐up phone calls at 6 h, 24 h, and 48 h postplacement, during which patients were reminded of upcoming doses if needed and asked to verbally confirm application/ingestion times. Nonadherent patients (e.g., those missing > 1 dose or unable to confirm timing) were excluded from the per‐protocol analysis, as detailed in the trial design. No automated messaging system was employed, as preliminary discussions with patients indicated a preference for direct phone contact to address any immediate questions.

The patients in the ketoprofen and diclofenac groups were asked to apply the transdermal patch on a hairless area of the skin such as the arm or forearm immediately after archwire placement and also after 12 and 24 h [26]. In the ibuprofen group, the patients were asked to take 400 mg ibuprofen tablets immediately after archwire placement and also after 8, 16, and 24 h. In addition, all patients received an envelope containing 10 acetaminophen tablets (500 mg) as the rescue drug to take in case their pain did not subside from the prescribed medication/patch. In addition, they were asked to inform the researcher in case of using the rescue drug. Patients using the rescue drug were excluded from the study and were visited at the shortest possible time to find the cause of their severe pain.

The patients were also requested to express their level of pain immediately after treatment, after 2 and 6 h, at bedtime, after 24 and 48 h, and also after 3 and 7 days following archwire placement using a numeric rating scale (NRS). Patients self‐reported their scores on a standardized, illustrated NRS form provided postplacement, which featured a 0–10 horizontal scale with verbal anchors (0 = No Pain; 10 = Worst Possible Pain Imaginable), pictorial facial expressions corresponding to each score level, and brief written instructions. They were guided to select and circle (or note) the number best reflecting their current pain intensity, with the researcher verbally confirming understanding and collecting responses via in‐person return at follow‐ups or phone/email at remote time points. They were asked to quantify their pain level by using a number between 0–10 that best described their experienced pain with 0 indicating no pain and 10 indicating the most excruciating pain possible. The NRS used in this study included both pictures and descriptions for an enhanced understanding [35].

### 2.4. Outcomes (Primary and Secondary)

The primary outcome was orthodontic pain severity, assessed using the NRS by patients at baseline (immediately postarchwire placement), 2 h, 6 h, bedtime (approximately 10–12 h postplacement), 24 h, 48 h, 3 days, and 7 days postplacement.

Secondary outcomes included (1) the trend of change in pain scores over time, analyzed via generalized estimating equations (GEEs) to compare longitudinal patterns across groups (unit: NRS score change from baseline), (2) the influence of demographic factors (age in years and gender as binary: male/female) on perceived pain, evaluated through GEE models assessing interaction effects (unit: NRS score adjusted for covariates), and (3) the incidence of adverse events (harms), such as gastrointestinal issues or skin reactions, reported as categorical events per group (unit: count and percentage of affected patients).

### 2.5. Sample Size Calculation

Sample size was calculated using PASS 11 (NCSS, Kaysville, UT, USA) for a one‐way ANOVA (superiority/difference design). Based on an effect size of *f* = 0.60, *α* = 0.05, and power (1 − *β*) = 0.95, the required sample size was estimated as 16 patients per group.

The assumptions for this calculation were informed by previously published pain data (mean = 2.59, SD = 1.56 from Farzanegan et al. [6]). Allowing for an anticipated 20% attrition, the target enrollment was increased to 20 patients per group (total *n* = 60).

*Note:* This sample size calculation corresponds to a superiority (difference) analysis using ANOVA. The study was therefore powered to detect medium–large between‐group differences rather than to formally establish noninferiority.

### 2.6. Interim Analyses and Stopping Guidelines

No interim analyses were performed, and no stopping guidelines were established.

### 2.7. Randomization

Using stratified permuted blocks, all eligible patients who consented to participate in the study randomly received an envelope containing the medication and instructions for use. The envelopes were administered by an assistant. Sixty patients underwent the randomization process, and their gender and type of medication were taken into account as the variables to randomly assign the patients into three groups (*n* = 20 with 10 males and 10 females in each group). The medications were also labeled A (ketoprofen), B (diclofenac), or C (ibuprofen). The envelopes were divided into two groups of blue and red. Female participants selected a red envelope, and male participants selected a blue envelope. The medications and their instructions for use were placed in the envelopes according to the following sequences:

 Blue envelope blocks: ABC, ACB, BAC, BCA, CBA, CAB, ABC, ACB, BAC, and BCA
 Red envelope blocks: CAB, CBA, ABC, ACB, BAC, BCA, ABC, ACB, CAB, and CBA

To ensure allocation concealment, sequentially numbered, sealed opaque envelopes were used [36].

### 2.8. Blinding

The orthodontist, researcher, and statistician were all blinded to the type of assigned medications to patients (their group allocation), and only the assistant who coded the envelopes was aware of their content. Due to the inherent differences between transdermal patches and oral tablets, blinding of participants to the mode of administration was not feasible; therefore, the study was open‐label with respect to the delivery method.

### 2.9. Statistical Analysis

Normal distribution of data was evaluated by the Kolmogorov–Smirnov test, and homogeneity of the variances was analyzed by Levene’s test. Quantitative variables (pain scores on the NRS and age in years) were analyzed by the GEE, Kruskal–Wallis test, and Bonferroni test, while qualitative variables (gender, treatment group, incidence of zero pain scores, and adverse events) were analyzed by the chi‐square or Fisher’s exact test. In the GEE models, an unstructured and an exchangeable working correlation matrix structure were used along with the Corrected Quasi‐Likelihood under Independence Model Criterion. All statistical analyses were carried out using SPSS Version 27 (SPSS Inc., IL, USA) at the 0.05 level of significance.

## 3. Results

### 3.1. Participant Flow

**FIGURE 1:**
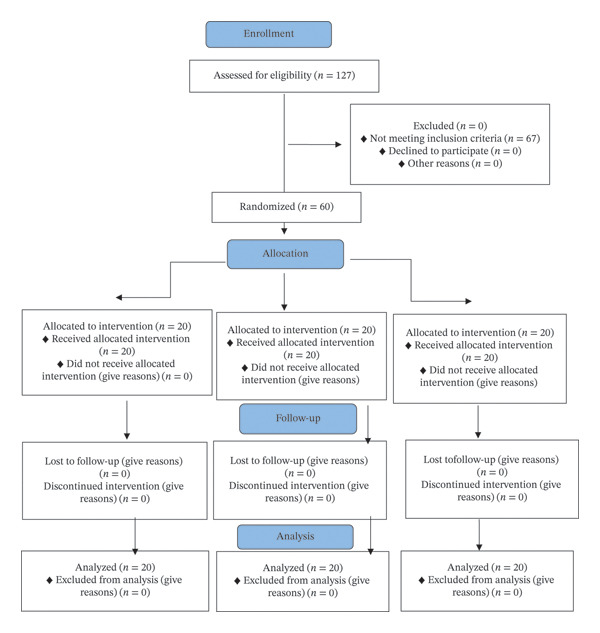
CONSORT flow diagram of patient selection and allocation.

The sample consisted of 60 patients with a mean age of 19.93 ± 4.64 years in three groups (Table 1). Each group included 10 males and 10 females. The mean age was not significantly different among the three groups (*p* = 0.972). Thus, the three groups were standardized in terms of age and gender. No patients were excluded due to adverse events, including the one case of gastrointestinal problems in the ibuprofen group (detailed in the Harms subsection), as it did not affect protocol adherence. Figure 1 shows the CONSORT flow diagram of patient selection and allocation.

**TABLE 1:** Demographic data of the patients.

Groups		Treatment group		
		Ketoprofen	Diclofenac	Ibuprofen
Age (years)		21.10 ± 5.06	18.95 ± 4.97	19.75 ± 3.88

Gender	Female	50% (10)	50% (10)	50% (10)
	Male	50% (10)	50% (10)	50% (10)

### 3.2. Harms

Only one patient developed gastrointestinal problems following the consumption of ibuprofen. Skin reactions or gastrointestinal problems were not observed in any patient in the ketoprofen or diclofenac group.

### 3.3. Subgroup Analyses

**FIGURE 2:**
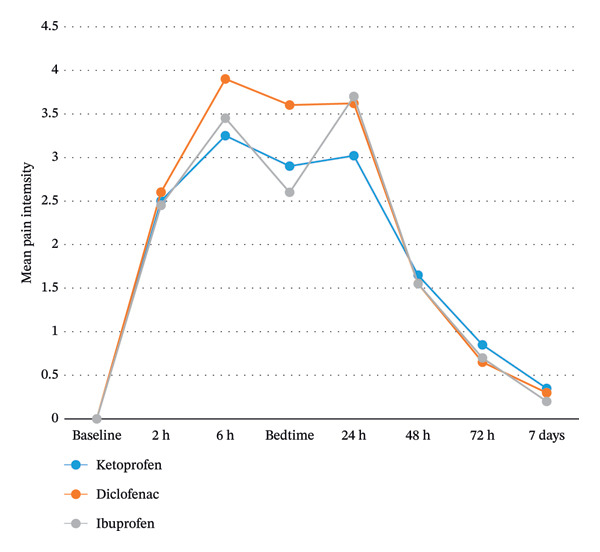
Trend of change in the pain score of orthodontic patients in the three groups.

Table 2 compares the pain score among the three groups at different time points following the placement of archwire. As shown, no significant difference was observed among the three groups in pain score at any time point (*p* > 0.05). In addition, in all three groups, no pain was observed at baseline. In all three groups, pain increased in the first 6 h and then decreased at bedtime. It increased again at bedtime until 24 h; this increase was greater in the ibuprofen group compared with the other two groups. Pain experienced a descending trend from 48 h to 7 days. GEE was applied to compare the trend of change in pain score among the three groups, which showed no significant difference (*p* > 0.05). The overall pain score was the lowest in the ketoprofen group, followed by the ibuprofen group, and then the diclofenac group; however, the difference did not reach statistical significance (Figure 2).

**TABLE 2:** Comparison of the pain score among the three groups at different time points following the placement of archwire.

Time	Treatment group			*p* value^∗∗^ (*F*)
	Ketoprofen mean ± SD	Diclofenac mean ± SD	Ibuprofen mean ± SD	
2 h	2.50 ± 1.64ab	2.60 ± 1.67ac	2.45 ± 1.47ad	0.857 (0.31)
6 h	3.25 ± 1.65a	3.90 ± 1.25b	3.45 ± 1.82bc	0.394 (1.86)
Bedtime	2.90 ± 1.80ab	3.60 ± 1.53ab	2.60 ± 1.79ab	0.202 (3.20)
24 h	3.02 ± 1.95a	3.62 ± 1.46ab	3.70 ± 1.81c	0.238 (2.87)
48 h	1.65 ± 1.39b	1.55 ± 1.23c	1.55 ± 1.23d	0.978 (0.04)
72 h	0.85 ± 1.23c	0.65 ± 0.93d	0.70 ± 0.66e	0.641 (0.89)
7 days	0.35 ± 0.49d	0.30 ± 0.66e	0.20 ± 0.41f	0.536 (1.25)
*p* value^∗^ (Wald Chi‐square)	< 0.001 (90.06)	< 0.001 (274.39)	< 0.001 (125.72)	

*Note:* Different lowercase letters in each column indicate the presence of a significant difference in pairwise comparisons of the time points (*p* > 0.05).

Abbreviation: SD, standard deviation.

^∗^GEE (Bonferroni pairwise comparison).

^∗∗^Kruskal–Wallis test.

Table 3 indicates the simultaneous effect of independent variables on the trend of pain score at the assessed time points. As indicated, only time had a significant effect on the trend of pain score (*p* < 0.001). Overall, male patients had a lower pain score than female patients; but this difference was not significant (*p* > 0.05).

**TABLE 3:** Simultaneous effect of independent variables on the trend of the pain score at the assessed time points.

Variable	Category	*B*	SE	Test statistic	*p* value^∗^
Group	Ketoprofen	−0.004	0.30	0.001	0.989
	Diclofenac	0.22	0.29	0.61	0.435
	Ibuprofen (R)	—	—	—	—

Gender	Female	0.35	0.24	2.04	0.153
	Male (R)	—	—	—	—

Age	—	−0.009	0.03	0.10	0.757

Time	2 h	2.23	0.21	111.71	< 0.001
	6 h	3.25	0.19	281.15	< 0.001
	Bedtime	2.75	0.20	184.89	< 0.001
	24 h	3.15	0.20	245.25	< 0.001
	48 h	1.30	0.14	88.69	< 0.001
	72 h	0.48	0.10	22.74	< 0.001
	7 days (R)	—	—	—	—

^∗^GEE; R, reference.

Table 4 compares the number of patients with zero pain score in the three groups. The results showed no significant difference in this regard among the three groups at any time point (*p* > 0.05).

**TABLE 4:** Comparison of the number of patients with zero pain score among the three groups.

Time	Ketoprofen (%) count	Diclofenac (%) count	Ibuprofen (%) count	*p* value
Baseline	20 (100%)	20 (100%)	20 (100%)	0.999^∗^
2 h	2 (10%)	3 (15%)	1 (5%)	0.877^∗^
6 h	1 (5%)	0 (0%)	1 (5%)	0.999^∗^
Bedtime	1 (5%)	0 (0%)	3 (15%)	0.625^∗^
24 h	2 (10%)	1 (5%)	1 (5%)	0.999^∗^
48 h	5 (25%)	4 (20%)	6 (30%)	0.819^∗∗^
72 h	9 (45%)	11 (55%)	8 (40%)	0.779^∗∗^
7 days	13 (65%)	16 (80%)	16 (80%)	0.819^∗∗^

^∗^Fisher’s exact test.

^∗∗^Chi‐square test.

## 4. Discussion

The present results showed that in the ketoprofen and ibuprofen groups, pain increased from 2 to 6 h after archwire placement, decreased at bedtime, increased again after 24 h, and then decreased from day 2 to day 7. An almost similar trend was also observed in the diclofenac group with the difference that the pain score remained unchanged at 24 h. The increased pain score in the early hours after using the patches may be due to the fact that cutaneous absorption of drugs, unlike oral intake or injection, reaches its peak usually after 30 min to 2 h, and is a gradual process. In addition, the plasma concentration of topical medications such as transdermal patches containing NSAIDs is significantly lower than oral NSAIDs, and a longer time is required to reach a constant therapeutic plasma level [37]. Moreover, local inflammation due to the application of orthodontic forces at different parts of the oral cavity can aggravate pain before the medications can exert their anti‐inflammatory effect [38].

The increased pain score in early hours after taking ibuprofen may be due to the time‐consuming nature of its gastrointestinal absorption [5]. Ibuprofen needs to be taken periodically to ensure its optimal analgesic efficacy since it has a half‐life of 1.8–2 h [39]. Pain reduction at bedtime in all three groups can indicate that the plasma concentration of medications reached the optimal level at bedtime [40]. It can also be due to the circadian rhythm since some inflammatory mediators such as interleukin‐6 and prostaglandin E2 decrease at night, lowering the degree of inflammation and subsequently the pain score at night. An increase in the level of melatonin at night can also affect pain perception since melatonin has anti‐inflammatory and antioxidant effects as well and can decrease pain by inhibiting the production of free radicals and inflammation. Furthermore, deep sleep decreases the activity of the sympathetic system and increases the activity of the parasympathetic system, potentially decreasing pain sensitivity [38, 41]. At 24 h, the pain score increased again, which was much higher in the ibuprofen group. The reason may be the constant plasma level of medications in the patch groups unlike the ibuprofen group [17]. Daily activities and mastication, and the increased cortisol level in the morning can be other reasons for the increased level of pain at 24 h [42]. The gradual reduction of pain in all three groups from day 2 to day 7 is probably due to the reduction of inflammation and progression of the healing process since acute inflammation in response to tissue damage usually reaches its peak within the first 24–48 h, and then, the activity of anti‐inflammatory factors such as interleukin‐10 increases, causing a reduction in the immune response and enhancement of healing, resulting in pain relief [43].

Compared to nonpharmacological methods for orthodontic pain relief, such as low‐level laser therapy (LLLT), chewing gum, bite wafers, and vibratory stimulation, the transdermal NSAID patches in our study provided reliable and sustained analgesia comparable to oral ibuprofen, with potentially fewer barriers to adherence. Recent randomized controlled trials and network meta‐analyses indicate that LLLT can reduce pain severity following archwire placement or separator use, often equivalently to ibuprofen [44, 45], though results are inconsistent, with some studies showing no superiority over placebo [46]. Chewing gum has demonstrated moderate efficacy in alleviating initial orthodontic pain, similar to paracetamol [3] or ibuprofen [6, 40] in certain trials, and a recent meta‐analysis supports its use as a low‐cost alternative [47]. Bite wafers offer pain relief akin to NSAIDs by promoting periodontal ligament stimulation [13], while vibratory devices yield mixed outcomes, with meta‐analyses concluding no overall significant reduction in pain [48]. Transdermal patches, by contrast, bypass gastrointestinal absorption issues associated with oral NSAIDs—evident in our single case of ibuprofen‐related adverse effects—and require no clinical equipment, repeated mechanical effort, or patient training, making them a convenient, patient‐preferred option for acute orthodontic pain management with minimal systemic exposure.

In the present study, pain was assessed using the NRS due to its superiority to the visual analog scale (VAS), enabling easier parametrical analysis [49]. The results showed that the mean pain score was 2.51 at 2 h, 3.53 at 6 h, 3.01 at bedtime, and 3.43 at 24 h. Following pain reduction starting from day 2, the mean pain score reached 0.28 at the end of the first week. These results were in line with the findings of a review study by Inauen et al. [38]. They evaluated 37 randomized clinical trial (2277 patients) regarding the pain score of patients at the leveling/alignment step using a 100‐mm VAS and reported a fast onset of pain right after archwire placement. The mean pain score was 22.98 after 2 h, 40.86 after 6 h, 29.85 after 8 h, and 42.42 after 24 h. It then started to gradually subside such that it reached 9 mm at the end of the first week. The trend of change in the pain score in their study was close to that in the present study. However, the pain score was lower at 6 and 24 h in the present study compared with theirs, which can be due to the regular intake and higher efficacy of medications used in the present study compared with pain control methods in other studies. In the current study, orthodontic pain was moderate (NRS between 3–6) on day 1, which was in line with the results of Olteanu et al. [41].

Literature is controversial regarding the effects of gender and age on perceived orthodontic pain. Olteanu et al. [41] showed a higher pain score in males than in females at the time of separator placement, cementation of bands, archwire activation, and elastic tension and increased with age. Unlike their study, the present results showed no significant effect of age and gender on the pain score. Similar results were reported by Ngan et al. [49] and Inauen et al. [38]. Thus, gender‐ or age‐related differences in pain perception may reflect cultural issues or personal experiences rather than actual physiological differences [50]. Additionally, factors such as dietary habits (e.g., consumption of hard or spicy foods that could exacerbate oral discomfort) were not controlled in our protocol, potentially contributing to interindividual variability in pain scores. The relatively modest sample size (*n* = 20 per group) and lack of division into finer age subgroups (e.g., adolescents vs. young adults) may have limited our power to detect subtle demographic interactions, underscoring the need for larger, stratified studies to explore these influences further.

In the present study, the pain score was the lowest in the ketoprofen group, followed by the ibuprofen group, and then the diclofenac group, but the difference was not significant. Thus, the null hypothesis of the study was accepted. The pain score was slightly, but not significantly, lower in the ketoprofen group than in the diclofenac group. Both of these medications are NSAIDs and decrease the prostaglandin synthesis by the inhibition of COX enzymes. In addition to the lower dosage of diclofenac than that of ketoprofen in the present study, these two medications have differences in selectivity for COX‐1 and COX‐2 enzymes, half‐life, and chemical formulation, which may affect their efficacy and safety [27, 51]. Diclofenac has a higher selectivity for COX‐2 and is therefore used for cases of chronic inflammation such as osteoarthritis; therefore, it may have a lower efficacy than ketoprofen for the management of orthodontic pain, which is due to acute inflammation, whereas ketoprofen may be more effective for faster relief of acute pains such as orthodontic pain due to better penetration into the inflamed tissues and its effect on the minor inflammatory pathways such as leukotriene inhibition [51]. In addition, diclofenac has a half‐life of 2 h, but in transdermal patches, the medications are released slowly, and the effects may remain for 12–24 h. Ketoprofen has a half‐life of up to 2.5 h, but due to its stronger attachment to plasma proteins and greater penetration into the inflamed tissues, its effects may last longer (up to 48 h) than those of diclofenac at the inflammation site [27]. Moreover, due to the polarity of its chemical structure, diclofenac has limited absorption through the skin, and carriers such as diethyl amine or fatty alcohols are often used to enhance its penetration. Thus, it has a slower absorption when applied through a transdermal patch. Ketoprofen has higher lipid solubility and can better penetrate deep into the tissues. It has a faster absorption through the skin and accumulates at the inflammation site in higher concentrations; these explanations justify the lower pain score in the ketoprofen group [28].

Karunakar et al. [27] evaluated the analgesic efficacy of diclofenac and ketoprofen patches and showed that they were both effective for management of postendodontic pain and can be used as an alternative to diclofenac tablets for this purpose. In addition, Dhanapal and Sureshbabu [29] suggested diclofenac transdermal patches as an alternative for the management of mild‐to‐moderate endodontic pain with fewer systemic side effects. Zadsirjan et al. [26] showed that ketoprofen transdermal patches had a comparable efficacy to ibuprofen tablets for postendodontic pain control in teeth with irreversible pulpitis. Furthermore, Mangal et al. [31] demonstrated comparable analgesic efficacy of the diclofenac patch and tablets for postendodontic pain control in single‐rooted premolars with irreversible pulpitis. Despite different methodologies, the abovementioned results were in line with the current findings, highlighting the optimal comparable analgesic efficacy of diclofenac and ketoprofen patches. The optimal comparable analgesic efficacy of diclofenac and ketoprofen patches for management of postextraction pain has also been reported [20, 24, 52].

In the present study, none of the patients in the patch groups experienced any gastrointestinal problem. In a study by Karunakar et al. [27], one out of 10 patients in the diclofenac patch group and none in the ketoprofen patch group developed gastrointestinal problems. Difference between their results and the present findings may be due to the administered dosage since the diclofenac patches in their study had 100 mg concentration (versus 15 mg in the present study). In the present study, no allergy or skin reaction occurred in any patient, which was similar to the study by Karunakar et al. [27]. However, in a study by Zadsirjan et al. [26], one patient had a skin reaction to the first dose of the ketoprofen patch, which may be due to the drug dosage (60 mg in their study versus 30 mg in the present study), their larger sample size (32 versus 20), and different patch structures due to different manufacturers. In the current study, no significant difference was found among the three groups in the frequency of patients with a pain score of zero. Similarly, Zadsirjan et al. [26] found no significant difference in this regard.

This study has several limitations. First, it lacked a placebo arm and participant blinding to the mode of administration; a placebo group was not included for ethical reasons. Because participants were aware whether they received a transdermal patch or an oral tablet, expectancy‐ or perception‐related (“novelty”) effects may have influenced subjective pain ratings. Although between‐group differences were not statistically significant, psychological contributions to the numerically lower scores observed in the ketoprofen patch group cannot be entirely excluded. Future trials should consider double‐dummy designs or, when ethically permissible, placebo controls to minimize such bias.

Second, the present trial was designed and powered as a superiority (difference) study. The sample size calculation was based on an ANOVA framework for detecting between‐group differences; therefore, the absence of a statistically significant difference should not be interpreted as evidence of noninferiority. Studies aiming to demonstrate noninferiority should prespecify an appropriate margin (Δ) and be adequately powered for that hypothesis.

Third, we used “bedtime” as the pain assessment time point and did not record exact clock times. As a result, the interval between medication administration and pain recording may have varied among participants; pharmacokinetic differences—particularly for drugs with shorter half‐lives—and circadian variation in pain perception may have affected reported pain intensity. Although such variability was likely nondifferential across randomized groups, future studies should standardize assessment timing (e.g., fixed‐hour intervals postplacement) or incorporate time interval as a covariate.

Finally, pain was assessed subjectively using self‐reported scales, and interindividual differences in pain perception may influence results. While NSAID intake during orthodontic treatment can affect tooth movement [53], medication in this study was limited to a single day, making clinically meaningful effects on tooth movement unlikely. Nonetheless, larger studies are needed to improve generalizability. Given the increasing use of clear aligners, future research should also evaluate pain control strategies in aligner patients compared with fixed‐appliance patients.

## 5. Conclusion

Considering the comparable analgesic efficacy of ibuprofen and the tested transdermal patches, diclofenac and ketoprofen patches may be used as an alternative to ibuprofen tablets for efficient pain control after initial archwire placement.

## Funding

This study was funded and supported by the Dental Sciences Research Center of Guilan University of Medical Sciences (5435).

## Disclosure

The authors acknowledge that a previous version of this manuscript was published as a preprint on the Authorea platform [54]. That version was posted during an earlier submission process and has not undergone peer review. The present version has been substantially revised and improved based on the updated data analysis and journal formatting and is submitted here for peer‐reviewed publication. This study is derived from the doctoral thesis of a postgraduate orthodontics student at Guilan University of Medical Sciences (IR.GUMS.REC.1402.577).

## Ethics Statement

This study was approved by the Ethics Committee of Guilan University of Medical Sciences (IR.GUMS.REC.1402.577). Written informed consent was obtained from all participants prior to enrollment.

## Conflicts of Interest

The authors declare no conflicts of interest.

## Data Availability

The data that support the findings of this study are available from the corresponding author upon reasonable request.

## References

[1] Ngan P. , Wilson S. , Shanfeld J. , and Amini H. , The Effect of Ibuprofen on the Level of Discomfort in Patients Undergoing Orthodontic Treatment, American Journal of Orthodontics and Dentofacial Orthopedics. (1994) 106, no. 1, 88–95, 10.1016/s0889-5406(94)70025-7, 2-s2.0-0028469182. 8017354

[2] Klasser G. D. and Okeson J. P. , Role of the Dentist in the Management of Orofacial Pain, Pain Management. (2015) 5, no. 6, 407–411, 10.2217/pmt.15.40, 2-s2.0-85050577679. 26400752

[3] Alshammari A. K. and Huggare J. , Pain Relief After Orthodontic Archwire Installation-A Comparison Between Intervention With Paracetamol and Chewing Gum: A Randomized Controlled Trial, The European Journal of Orthodontics. (2019) 41, no. 5, 478–485, 10.1093/ejo/cjy081, 2-s2.0-85072513832. 30590573

[4] Eslamian L. , Torshabi M. , Motamedian S. R. , Hemmati Y. B. , and Mortazavi S. A. , The Effect of Naproxen Patches on Relieving Orthodontic Pain by Evaluation of VAS and IL-1β Inflammatory Factor: A Split-Mouth Study, Dental Press Journal of Orthodontics. (2019) 24, no. 6, 27e1–27e7, 10.1590/2177-6709.24.6.27.e1-7.onl. 31994643 PMC6986181

[5] Sigaroodi A. K. , Motevasseli S. , Maleki D. , Maleki D. , and Fard R. S. , Low-Level Laser and Management of Common Complications After the Mandibular Third Molar Surgery: A Double-Blind Randomized Clinical Trial, Dental Research Journal. (2023) 20, no. 1, 10.4103/1735-3327.367913. PMC9937931 36820144

[6] Farzanegan F. , Zebarjad S. M. , Alizadeh S. , and Ahrari F. , Pain Reduction After Initial Archwire Placement in Orthodontic Patients: A Randomized Clinical Trial, American Journal of Orthodontics and Dentofacial Orthopedics. (2012) 141, no. 2, 169–173, 10.1016/j.ajodo.2011.06.042, 2-s2.0-84856296684. 22284284

[7] Kanzaki H. , Chiba M. , Shimizu Y. , and Mitani H. , Periodontal Ligament Cells Under Mechanical Stress Induce Osteoclastogenesis by Receptor Activator of Nuclear Factor KappaB Ligand Up-Regulation via Prostaglandin E2 Synthesis, Journal of Bone and Mineral Research. (2002) 17, no. 2, 210–220, 10.1359/jbmr.2002.17.2.210, 2-s2.0-0036157354. 11811551

[8] Roth P. M. and Thrash W. J. , Effect of Transcutaneous Electrical Nerve Stimulation for Controlling Pain Associated With Orthodontic Tooth Movement, American Journal of Orthodontics and Dentofacial Orthopedics. (1986) 90, no. 2, 132–138, 10.1016/0889-5406(86)90045-4, 2-s2.0-0022759231. 3488674

[9] Stanfeld J. , Jones J. , Laster L. , and Davidovitch Z. , Biochemical Aspects of Orthodontic Tooth Movement. I. Cyclic Nucleotide and Prostaglandin Concentrations in Tissues Surrounding Orthodontically Treated Teeth In Vivo, American Journal of Orthodontics and Dentofacial Orthopedics. (1986) 90, no. 2, 139–148. 3017094 10.1016/0889-5406(86)90046-6

[10] Naesdal J. and Brown K. , NSAID-Associated Adverse Effects and Acid Control Aids to Prevent Them: A Review of Current Treatment Options, Drug Safety. (2006) 29, no. 2, 119–132. 16454539 10.2165/00002018-200629020-00002

[11] Hersh E. V. , Moore P. A. , Grosser T. et al., Nonsteroidal Anti-Inflammatory Drugs and Opioids in Postsurgical Dental Pain, Journal of Dental Research. (2020) 99, no. 7, 777–786, 10.1177/0022034520914254. 32286125 PMC7313348

[12] Sharma A. A. , Jadhav A. A. , Bhola N. D. , Gupta A. A. , and Gupta C. S. , Pre-Emptive Analgesic Efficacy of Single-Dose Transdermal Ketoprofen and Diclofenac Patches in Post-Operative Pain Management Following Open Treatment of Mandibular Fractures: A Randomized Controlled Study, Cureus. (2022) 14, no. 8, 10.7759/cureus.27982. PMC9468632 36120190

[13] Hernandez C. , Emer J. , and Robinson J. K. , Perioperative Management of Medications for Psoriasis and Psoriatic Arthritis: A Review for the Dermasurgeon, Dermatologic Surgery. (2008) 34, no. 4, 446–459, 10.1111/j.1524-4725.2007.34091.x, 2-s2.0-41149176165. 18248470

[14] Mills S. B. , Bloch M. , and Bruckner F. E. , Double-Blind Cross-Over Study of Ketoprofen and Ibuprofen in Management of Rheumatoid Arthritis, British Medical Journal. (1973) 4, no. 5884, 82–84, 10.1136/bmj.4.5884.82, 2-s2.0-0015821569. 4583184 PMC1587224

[15] Bachalli P. S. , Nandakumar H. , and Srinath N. , A Comparative Study of Diclofenac Transdermal Patch Against Oral Diclofenac for Pain Control Following Removal of Mandibular Impacted Third Molars, Journal of Maxillofacial and Oral Surgery. (2009) 8, no. 2, 167–172, 10.1007/s12663-009-0041-8, 2-s2.0-84908029807. 23139499 PMC3453945

[16] Shinde V. A. , Kalikar M. , Jagtap S. et al., Efficacy and Safety of Oral Diclofenac Sustained Release Versus Transdermal Diclofenac Patch in Chronic Musculoskeletal Pain: A Randomized, Open-Label Trial, Journal of Pharmacology and Pharmacotherapeutics. (2017) 8, no. 4, 166–171, 10.4103/jpp.jpp_35_17, 2-s2.0-85041661654. 29472748 PMC5820746

[17] Wang L. , Ma J. , Li J. , Fang L. , and Liu C. , Transdermal Patch Based on Pressure-Sensitive Adhesive: The Importance of Adhesion for Efficient Drug Delivery, Expert Opinion on Drug Delivery. (2025) 22, no. 3, 405–420, 10.1080/17425247.2025.2460650. 39881563

[18] Bhargava D. , Thomas S. , and Beena S. , Comparison Between Efficacy of Transdermal Ketoprofen and Diclofenac Patch in Patients Undergoing Therapeutic Extraction—A Randomized Prospective Split-Mouth Study, Journal of Oral and Maxillofacial Surgery. (2019) 77, no. 10, 1998–2003, 10.1016/j.joms.2019.04.007, 2-s2.0-85065850116. 31077671

[19] Krishnan S. , Sharma P. , Sharma R. , Kumar S. , Verma M. , and Chaudhary Z. , Transdermal Diclofenac Patches for Control of Post-Extraction Pain: A Pilot Randomized Controlled Double-Blind Study, Oral and Maxillofacial Surgery. (2013) 19, no. 1, 5–12, 10.1007/s10006-013-0422-5, 2-s2.0-84879860687. 23852077

[20] Pandey K. , Shettar V. , and Kale T. , Efficacy of Transdermal Ketoprofen Patch in Comparison to Transdermal Diclofenac Patch in Postoperative Analgesia for Orthodontic Extractions: A Randomized Split-Mouth Study, Cureus. (2023) 15, no. 4, 10.7759/cureus.37732. PMC10191808 37213950

[21] Kalita F. , Gehlot N. , Gupta D. S. , and Mitra S. , Transdermal Patches: Do They Really Work?—A Comparative Study of Ketoprofen Versus Diclofenac Analgesic Patches in Minor Oral Surgery, Oral Surgery. (2024) 17, no. 2, 106–112, 10.1111/ors.12850.

[22] Kripal K. , Bm C. , Chandrasekaran K. , and Dileep A. , Comparative Evaluation of Analgesic Efficacy of Oral Diclofenac Sodium and Transdermal Patch (Nupatch®) After Periodontal Flap Surgery: A Randomized Crossover Clinical Trial, International Journal of Scientific Research. (2019) 7, no. 11, 27–29.

[23] Diwan V. , Srinivasa T. S. , Ramreddy K. Y. , Agrawal V. , Nagdeve S. , and Parvez H. , A Comparative Evaluation of Transdermal Diclofenac Patch With Oral Diclofenac Sodium as an Analgesic Drug Following Periodontal Flap Surgery: A Randomized Controlled Clinical Study, Indian Journal of Dental Research. (2019) 30, no. 1, 57–60, 10.4103/ijdr.IJDR_84_17. 30900658

[24] Murthykumar K. and Varghese S. , Analgesic Efficacy and Safety of Transdermal and Oral Diclofenac in Postoperative Pain Management Following Periodontal Flap Surgery, Drug Invention Today. (2019) 11, 652–656.

[25] Jadhav P. , Sinha R. , Uppada U. K. , Tiwari P. K. , and Avss S. K. , Pre-Emptive Diclofenac Versus Ketoprofen as a Transdermal Drug Delivery System: How They Face, Journal of Maxillofacial and Oral Surgery. (2017) 17, no. 4, 488–494, 10.1007/s12663-017-1048-1, 2-s2.0-85072151765. 30344391 PMC6181849

[26] Zadsirjan S. , Toghrolian A. , and Zargar N. , Analgesic Efficacy of Ketoprofen Transdermal Patch Versus Ibuprofen Oral Tablet on Postendodontic Pain in Patients With Irreversible Pulpitis: A Randomized Clinical Trial, Pain Research and Management. (2023) 2023, 8549655–10, 10.1155/2023/8549655. 37324280 PMC10266914

[27] Karunakar P. , Reddy M. , Karteek B. , Reddy C. L. C. , Swetha C. , and Racha K. , Comparative Evaluation of Efficacy of Diclofenac and Ketoprofen Administered Using Transdermal Drug Delivery Route in Management of Post Endodontic Pain: A Randomized Controlled Clinical Trial, Journal of Conservative Dentistry and Endodontics. (2024) 27, no. 1, 24–28, 10.4103/jcde.jcde_160_23. 38389736 PMC10880480

[28] Porwal P. , Shah N. , Rao A. S. et al., Comparative Evaluation of Efficacy of Ketoprofen and Diclofenac Transdermal Patches With Oral Diclofenac Tablet on Postoperative Endodontic Pain—A Randomized Clinical Trial, Patient Preference and Adherence. (2023) 17, 2385–2393, 10.2147/PPA.S421371. 37790865 PMC10544139

[29] Dhanapal S. and Sureshbabu N. M. , Efficacy of Single Dose of Transdermal Patch as a Pre-Operative Analgesic in Root Canal Treatment—A Randomized Clinical Trial, Journal of Pharmaceutical Sciences and Research. (2016) 8, no. 2.

[30] Bhaskar H. , Kapoor P. , and Ragini , Comparison of Transdermal Diclofenac Patch With Oral Diclofenac as an Analgesic Modality Following Multiple Premolar Extractions in Orthodontic Patients: A Crossover Efficacy Trial, Contemporary Clinical Dentistry. (2010) 1, no. 3, 158–163, 10.4103/0976-237x.72783. 22114407 PMC3220102

[31] Mangal S. , Mathew S. , Murthy B. V. , Hegde S. , Dinesh K. , and Ramesh P. , The Efficacy of Transdermal and Oral Diclofenac for Post-Endodontic Pain Control: A Randomized Controlled Trial, Indian Journal of Dental Research. (2020) 31, no. 1, 53–56, 10.4103/ijdr.IJDR_167_17. 32246682

[32] Little R. M. , The Irregularity Index: A Quantitative Score of Mandibular Anterior Alignment, American Journal of Orthodontics. (1975) 68, no. 5, 554–563, 10.1016/0002-9416(75)90086-x, 2-s2.0-0016571462. 1059332

[33] Angelopoulou M. V. , Vlachou V. , and Halazonetis D. J. , Pharmacological Management of Pain During Orthodontic Treatment: A Meta-Analysis, Orthodontics and Craniofacial Research. (2012) 15, no. 2, 71–83, 10.1111/j.1601-6343.2012.01542.x, 2-s2.0-84860151073. 22515183

[34] Farzanegan F. , Bamrood Z. A. , Rangrazi A. , and Shafaee H. , Effect of Ibuprofen and Low-Intensity Pulsed Ultrasound on the Reduction of Pain After Initial Archwire Placement: A Double-Blind Randomized Clinical Trial, Journal of Oral Research. (2022) 11, no. 1, 1–12, 10.17126/joralres.2022.001.

[35] Price D. D. , Patel R. , Robinson M. E. , and Staud R. , Characteristics of Electronic Visual Analogue and Numerical Scales for Ratings of Experimental Pain in Healthy Subjects and Fibromyalgia Patients, Pain. (2008) 140, no. 1, 158–166, 10.1016/j.pain.2008.07.028, 2-s2.0-54249103985. 18786761

[36] Doig G. S. and Simpson F. , Randomization and Allocation Concealment: A Practical Guide for Researchers, Journal of Critical Care. (2005) 20, no. 2, 187–191, 10.1016/j.jcrc.2005.04.005, 2-s2.0-24044457559. 16139163

[37] Modi K. , Sundar S. , Kowsky D. , and Natanasabapathy V. , Diclofenac Transdermal Patch Versus Oral Diclofenac on Post-Endodontic Pain and Quality of Life: A Randomized Clinical Trial, European Endodontic Journal. (2023) 9, no. 3, 203–209. 10.14744/eej.2024.37233 PMC11413605 38619496

[38] Inauen D. S. , Papadopoulou A. K. , Eliades T. , and Papageorgiou S. N. , Pain Profile During Orthodontic Levelling and Alignment With Fixed Appliances Reported in Randomized Trials: A Systematic Review With Meta-Analyses, Clinical Oral Investigations. (2023) 27, no. 5, 1851–1868, 10.1007/s00784-023-04931-5. 36879148 PMC10159949

[39] Al-Dalaen S. M. , Hamad A. W. , AL-Hujran T. A. et al., Bioavailability and Bioequivalence of Two Oral Single Doses of Ibuprofen 400 mg to Healthy Volunteers, Biomedical and Pharmacology Journal. (2021) 14, no. 1, 435–444, 10.13005/bpj/2143.

[40] Alshayea E. , Aldweesh A. , Albalbeesi H. , and Aldosari M. , Comparative Assessment Between Chewing Gum, Bite Wafers, and Ibuprofen in Pain Control Following Separators Placement Among Orthodontic Patients, Saudi Dental Journal. (2024) 36, no. 7, 1010–1014, 10.1016/j.sdentj.2024.04.010. 39035560 PMC11255942

[41] Olteanu C. D. , Bucur S. M. , Chibelean M. , Bud E. S. , Păcurar M. , and Feștilă D. G. , Pain Perception During Orthodontic Treatment With Fixed Appliances, Applied Sciences. (2022) 12, no. 13, 10.3390/app12136389.

[42] Alturki G. , Jamel A. , Alshuaybi A. , Baeshen H. , and Farag A. M. , Perception of Pain Intensity and Quality in Patients Treated With Conventional Fixed Orthodontic Appliances Versus Clear Removable Aligners: A Pilot Study, The Open Dentistry Journal. (2024) 18, no. 1, 10.2174/0118742106314583240801074709.

[43] Hosseinzadeh P. , Karimi Nasab N. , Kalantar R. , and Maleki D. , COVID-19 and Orthodontic Emergencies: A Narrative Review, Journal of Dentomaxillofacial Radiology, Pathology and Surgery. (2021) 10, no. 4, 1–5.

[44] Bicakci A. A. , Kocoglu-Altan B. , Toker H. , Mutaf I. , and Sumer Z. , Efficiency of Low-Level Laser Therapy in Reducing Pain Induced by Orthodontic Forces, Photomedicine and Laser Surgery. (2012) 30, no. 10, 683–688, 10.1089/pho.2012.3245, 2-s2.0-84864958312. 22775467

[45] AlSayed H. M. M. A. , Mohamed M. N. S. , Hameed M. S. et al., Comparative Efficacy of Ibuprofen and Low-Level Laser Therapy on Pain Control Following Orthodontic Elastomeric Separator Placement: A Bayesian Network Meta-Analysis, Medicine (Baltimore). (2025) 104, no. 30. 10.1097/MD.0000000000043559 PMC12303510 40725907

[46] Dornelles-Mainardi C. , Fraccalini M. G. , Pinzan-Vercelino C. R. M. et al., Effect of Low-Level Laser Therapy on Pain Resulting From Orthodontic Interproximal Stripping in Adolescents: A Randomized Triple-Blind Clinical Trial, Lasers in Medical Science. (2025) 40, no. 1.

[47] Li J. , Yang X. , Xia Y. et al., Effect of Chewing Gum on Orthodontic Pain in Patients Receiving Fixed Orthodontic Treatment: A Systematic Review and Meta-Analysis, European Journal of Medical Research. (2023) 28, no. 1. 10.1186/s40001-023-01467-y PMC10631172 37936237

[48] Kalra S. , Kalra S. , and Kaur M. , The Effect of Vibrational Devices on Pain in Patients Undergoing Orthodontic Treatment: A Systematic Review and Meta-Analysis, The Journal of Indian Orthodontic Society. (2023) 57, no. 2, 115–123.

[49] Bolton J. E. and Wilkinson R. C. , Responsiveness of Pain Scales: A Comparison of Three Pain Intensity Measures in Chiropractic Patients, Journal of Manipulative and Physiological Therapeutics. (1998) 21, no. 1, 1–7. 9467094

[50] Ngan P. , Kess B. , and Wilson S. , Perception of Discomfort by Patients Undergoing Orthodontic Treatment, American Journal of Orthodontics and Dentofacial Orthopedics. (1989) 96, no. 1, 47–53, 10.1016/0889-5406(89)90228-x, 2-s2.0-0024706738. 2750720

[51] Bergius M. , Kiliaridis S. , and Berggren U. , Pain in Orthodontics: A Review and Discussion of the Literature, Journal of Orofacial Orthopedics. (2000) 61, no. 2, 125–137, 10.1007/bf01300354, 2-s2.0-0033657647. 10783564

[52] Kapo S. M. , Rakanović-Todić M. , Burnazović-Ristić L. et al., Analgesic and Anti-inflammatory Effects of Diclofenac and Ketoprofen Patches in Two Different Rat Models of Acute Inflammation, Journal of King Saud University – Science. (2023) 35, no. 1.

[53] Shankar D. , Sinha A. , Anand S. , Verma N. , and Choudhary S. , Efficacy of Transdermal Diclofenac Patch and Ketoprofen Patch as Postoperative Analgesia After Extraction of First Premolars Bilaterally in Both Arches for Orthodontic Purposes: A Comparative Study, Journal of Pharmacy and BioAllied Sciences. (2021) 13, no. Suppl 1, S101–S104, 10.4103/jpbs.jpbs_571_20. 34447053 PMC8375963

[54] Qanita A. A. and Orienty F. N. , Effect of Analgesic Medications Taken by Orthodontic Patients on Tooth Movement, Makassar Dental Journal. (2024) 13, no. 2, 312–318.

[55] Babaee Hemmati Y. and Toghrolian A. , Analgesic Efficacy of Diclofenac and Ketoprofen Patches Versus Ibuprofen Tablets for Pain Control After Archwire Placement in Orthodontic Patients, Authorea. (2025) 10.22541/au.174582202.28212902/v1. PMC13195437 42170735

